# Three-dimensional craniofacial imaging in children with achondroplasia treated with vosoritide

**DOI:** 10.1016/j.gimo.2025.103463

**Published:** 2025-10-13

**Authors:** Hanne Hoskens, J.David Aponte, Cassidy Da Silva, Trevor Coward, Benedikt Hallgrímsson, Melita Irving

**Affiliations:** 1 Department of Cell Biology and Anatomy, Cumming School of Medicine, University of Calgary, Calgary, AB, Canada; 2 Alberta Children's Hospital Research Institute, Cumming School of Medicine, University of Calgary, Calgary, AB, Canada; 3 Department of Craniofacial Sciences, King’s College London, London, United Kingdom; 4 Academic Centre of Reconstructive Science, Kings College London, United Kingdom; 5 Department of Clinical Genetics, Guy’s and St Thomas’ NHS Trust, London, United Kingdom

**Keywords:** Achondroplasia, Facial imaging, Geometric morphometrics, Vosoritide

## Abstract

**Purpose:**

Achondroplasia is the most common form of disproportionate short-stature skeletal dysplasia. Constitutively activated FGFR3 signaling disrupts endochondral ossification, affecting long bone growth, as well as the craniofacial skeleton and skull base. This results in the distinctive craniofacial features of achondroplasia, such as midface hypoplasia and nasal bridge depression. Vosoritide is a precision medicine that acts through the C-type natriuretic peptide pathway to counteract overactive FGFR3 signaling, including its inhibitory effects on chondrocyte differentiation and proliferation. The aim of this study was to investigate the potential effects of vosoritide treatment on the craniofacial skeleton in achondroplasia.

**Methods:**

We used longitudinal 3D facial surface imaging in children aged 5 to 10 years receiving vosoritide to test for differences in face shape over the course of treatment. We used nonlinear registration to standardize a dense measurement of all facial meshes. Finally, we defined the achondroplasia phenotype by scoring participant faces based on their shape similarity to the distinctive facial presentation of achondroplasia.

**Results:**

We measured the change in achondroplasia score for all participants recruited in this study. Using a linear mixed model approach to account for individual differences, we detected a small but significant reduction in the achondroplasia phenotype score over the course of vosoritide treatment.

**Conclusion:**

Our findings indicate that vosoritide treatment leads to a measurable improvement in facial phenotype in achondroplasia, highlighting its potential utility beyond enhancing linear growth. We discuss the role of pathway-disrupting medications, such as vosoritide, in addressing the broader spectrum of medical issues experienced by children with achondroplasia.

## Introduction

Achondroplasia (OMIM 100800) is the most common of the skeletal dysplasia conditions, a heterogeneous group of 771 different types of genetic disorders impacting the bones, joints, and skeleton.^1^ Affecting around 1 in 25,000 live births,^2^ it is caused by a recurrent pathogenic variant in the gene that includes fibroblast growth factor receptor type 3 (*FGFR3*), Gly380Arg.^3^^,^^4^ This results in constitutively activated FGFR3 signaling through a number of pathways, including the mitogen-activated protein kinase (MAPK) pathway, which ultimately inhibits endochondral ossification at the cartilaginous growth plates.^5^

Achondroplasia is characterized clinically by midface hypoplasia caused by an underdeveloped cranial base due to inhibition of endochondral ossification. This causes a retruded midface appearance, which can contribute to dental malocclusion and sleep apnea in 56% of people with achondroplasia.^6^ The underdeveloped cartilaginous bones of the midface also result in a concave nasal bridge and a short anteverted nose. The underdeveloped cranial base often leads to foramen magnum stenosis, which increases the risk of spinal cord impingement.^7^^,^^8^ Finally, achondroplasia is frequently associated with macrocephaly and frontal bossing, which are caused by disproportionate growth of the cranial vault in relation to the midface.

Vosoritide is a synthetic analog of C-type natriuretic peptide (CNP) that works to counteract constitutively activated FGFR3 and the downstream MAPK pathway activity that causes achondroplasia. Loss of CNP and its receptor natriuretic peptide receptor 2 has been shown to cause dwarfism in mice, whereas overexpression of CNP has been shown to promote endochondral ossification.^5^ This crosstalk between natriuretic peptide receptor 2 and MAPK alleviates the disruption to endochondral bone growth.7 In achondroplasia mouse models, vosoritide treatment significantly improves bone growth, corrects growth-plate defects, and reduces dwarfism-related skeletal morphologies, including notable changes to the skull.^9^^,^^10^

In the clinical trial setting, vosoritide has been shown to increase height in children with achondroplasia because of its effects on long bone growth.^11^^,^^12^ The effects are seen across all age groups, including those treated under the age of 5 years.^13^ It is this age group in which some of the most severe complications of achondroplasia are observed, namely, compression of the spinal cord at the craniocervical junction and sleep-disordered breathing caused by the effects on the facial bones that form the nasopharyngeal space.^14^, ^15^, ^16^ Consequently, there is a 50-fold increase in sudden infant death among infants with achondroplasia.^17^

Best practice recommendations exist to guide clinical management in the multidisciplinary setting across multiple specialties and over all age groups,^18^ with vosoritide being the only approved medical treatment designed to address the underlying molecular cause of achondroplasia.^19^

We aimed to explore the potential effects of vosoritide upon the more severe medical complications of achondroplasia, which are caused by its impact on the bones of the craniofacial skeleton.

## Materials and Methods

### Participants

**Figure 1:**
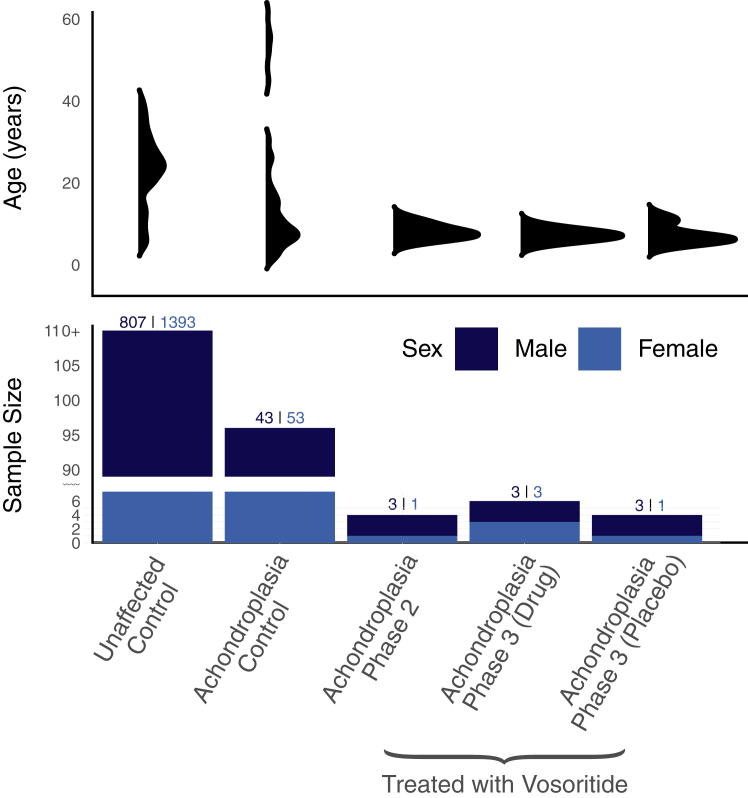
**Age and Sex distribution for all data sets included.**

A total of 14 individuals aged 5 to 10 years with achondroplasia were recruited at the Evelina London Children’s Hospital as part of the vosoritide phase 2 and phase 3 clinical trial program, BMN111-202/205 and BMN111-301/302. All participants had a confirmed molecular diagnosis. Phase 2 individuals (*N* = 4) all received the drug. Beginning with their first visit, phase 3 participants were randomly assigned to receive either the placebo (*N* = 4) or the drug (*N* = 6), with the placebo group receiving the drug 1 year later. As a result, the treated group consists of several treatment programs: phase 2, phase 3 drug, and phase 3 placebo. All phase 2 and phase 3 participants receiving the drug were treated with daily doses of 15 μg/kg of vosoritide. For each individual in the treated group, we obtained 3D facial surface images and demographic data (age, sex) before treatment and at successive follow-up visits (*N* = 142 images in total). This work thus represents a secondary use of the originally published phase 2 and 3 trials that estimated the effects of vosoritide on growth trajectories.^12^^,^^13^ Figure 1 illustrates the different data sets broken down by age and sex. Supplemental Table 1 contains more detailed information about the participants’ ages at each visit, ages at first dose administered, treatment groups, and measured achondroplasia scores.

### Controls

A total of 35 achondroplasia controls, who did not receive any treatment, were recruited through the multidisciplinary achondroplasia clinic at the Evelina London Children’s Hospital and served as a reference cohort in combination with 61 achondroplasia scans collected through an existing FaceBase data set (FaceBase Accession ID: https://doi.org/10.25550/TJ0; ^20^). A second cohort consisting of unaffected, normative controls (*N* = 2200) was sampled from the 3D Facial Norms study (FaceBase Accession ID: https://doi.org/10.25550/VWP^21^; Figure 1).

### 3D facial phenotyping

The Canfield Scientific Vectra H1 3D Imaging System was used to capture 3D facial surface images from treated participants, as well as achondroplasia controls at Evelina London Children’s Hospital. The facial surfaces of normative and achondroplasia controls from FaceBase were captured using the 2-pod 3dMDface or multipod 3dMDcranial systems. We nonrigidly registered an average facial atlas to each 3D image using the MeshMonk pipeline^22^ to obtain standard facial representations defined by 5629 homologous points. This was performed on the original images and their bilateral reflected copies. For each participant, symmetric configurations were computed as the average of the original and reflected image after Procrustes superimposition.

Images were visually inspected and excluded if they were of poor quality (eg, nonneutral facial expression, hair obscuring facial surface) or if the registration process had failed. The final analysis sample included all 14 participants from the treated group (*N* = 95 images in total) and 96 achondroplasia controls.

Finally, faces of the achondroplasia (treated and controls) and normative samples were aligned and scaled to unit size by generalized Procrustes analysis. We applied principal component analysis (PCA) to describe the major modes of shape variation and used parallel PCA to determine the number of principal components to use in the study. From that analysis, 50 PCs that collectively explained 97.8% of the total shape variation were retained. This step also acted as a quality control measure because later components typically represent noise in the data.

### Achondroplasia facial gestalt and variation in presentation

To delineate the common facial features of achondroplasia, we fitted a regression model for the cubic effect of age, sex, and group to contrast achondroplasia control and normative morphology.^23^ The phenotypic score, or the degree to which an individual displays facial features characteristic of achondroplasia, was measured by scalar projection of each participant onto the vector coefficient for the group variable, ie, the vector spanning normative and achondroplasia control shape means, accounting for age and sex effect.^24^ Phenotype scores were normalized by dividing by the norm of the established shape vector. Faces with increasingly characteristic or exaggerated features of achondroplasia controls scored higher on this axis, whereas faces that were more similar to the unaffected control face score lower.

### Statistical analysis

We tested for significant differences in total facial shape in the treated group between the first and last visit using Hotelling’s T2 statistic. Participants from both phase 2 and phase 3 were grouped together to increase sample size; none of them had received the drug during the initial visit, with all of them having received multiple doses by the final visit. Because of the sample size, we restricted Hotelling’s T2 tests to the first 13 principal component axes, which accounted for 85.6% of the total variation in face shape.

To evaluate the effect of the vosoritide on achondroplasia score across successive follow-up visits, we fitted a linear mixed model (lmer function, lme4 package in R) to the treated group (phase 2 and 3 combined). We modeled the fixed effects of treatment protocol (ie, placebo before drug administration vs drug administration throughout) and years from first treatment, as well as their interaction. “Individual” was treated as a random effect to account for individual differences in phenotypic score, before receiving vosoritide.

To disentangle the effects of vosoritide (which is measured over time from the start of treatment) from the natural effects of aging on facial shape, and potentially on phenotypic score, we modeled the effects of age in the achondroplasia control group (who did not receive any treatment) using linear regression (function lm, stats package in R). If a significant age effect was found, scores of treated participants were standardized based on the regression coefficients obtained from the achondroplasia control group before applying the mixed linear model approach described above.

### Code availability

All of the code used to preprocess and analyze the data for this study is publicly available on Github at https://github.com/Hallgrimsson-lab/vosoritideAchondroplasia. This includes the steps for symmetrization of faces, alignment and PCA of shape data, regression modeling to create the achondroplasia shape axis and score individuals, and statistical tests. We additionally include the code used to create the figures for this manuscript.

## Results

### Assessment of facial features and variation in achondroplasia

**Figure 2:**
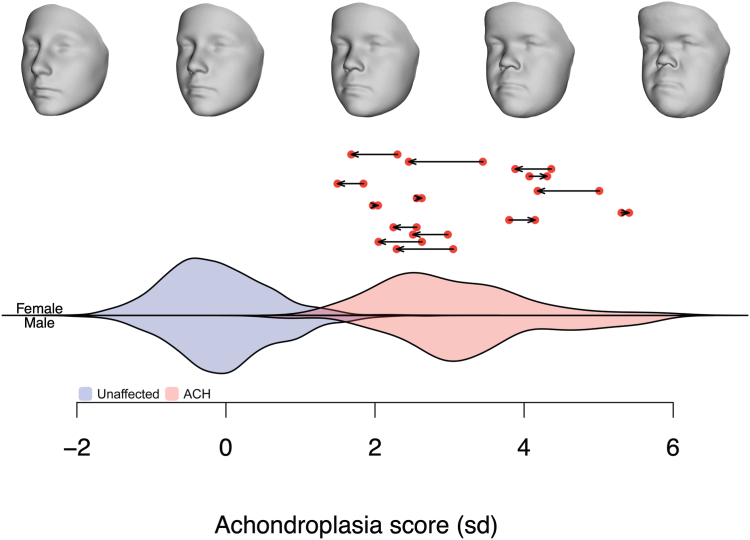
**Axis of variation between unaffected controls and achondroplasia controls.** The top row depicts the change in phenotype observed as achondroplasia score increases. Score distributions for the unaffected (blue) and achondroplasia (red) controls are expressed in standard deviations of achondroplasia control scores. The red dots with arrows show the change in score from first visit to last visit for the 14 participants in the study.

The axis of variation defined by the difference between age and sex-adjusted normative controls and achondroplasia controls is displayed at the top of Figure 2 as a series of morphs of increasing similarity to the achondroplasia mean face. The progression of morphs highlights the characteristic craniofacial features such as macrocephaly with frontal bossing, midfacial hypoplasia, and a depressed nasal bridge. The extent to which these features were present varied for individual participants. By contrasting the achondroplasia mean face to a comparable age- and sex-adjusted normative average, we established a shape axis that captured the range of facial features observed. A phenotypic score for each participant was subsequently expressed in a quantitative manner by projecting them onto this axis. The distribution of the scores for the achondroplasia controls (red) and normative controls (blue) is shown in Figure 2. The change in scores for the achondroplasia treated sample from first visit to last visit are displayed with arrows above the distributions. The participants recruited for this study showed substantial variation in achondroplasia scores before the administration of vosoritide. This variation was consistent with the expressivity of the achondroplasia phenotype in achondroplasia controls. The mildest achondroplasia individuals overlapped with nonsyndromic individuals who most closely resemble the achondroplasia phenotype, often normative controls with prominent brows and mildly retruded midface morphology.

### Evaluation of drug treatment on facial shape and phenotype score

**Figure 3:**
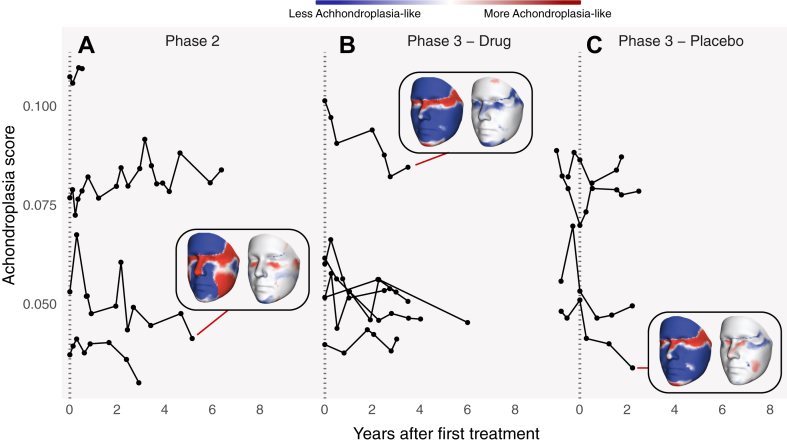
**Achondroplasia score at each visit for each participant, shown by study phase.** Participants in phase 3 placebo (rightmost panel) were measured several times over a 1 year period before beginning vosoritide treatment. For each phase, we show a heatmap for the participant with largest score change. The left heatmap shows the face at the first visit as a heatmap of achondroplasia scores, with blues signifying regions of the face that are more similar to unaffected controls, and reds signifying similarity to the achondroplasia phenotype. The right heatmap shows the difference in score at the last visit. Regions in blue score more “unaffected” at the last visit, whereas regions in red score higher for achondroplasia at the last visit.

To test for changes in full face shape among treated participants, we compared the paired first and last scan for all participants given vosoritide and found no significant difference (F_(13,1)_ = .7358, *P* = .7353). We then investigated whether we could detect a change in achondroplasia scores across all individuals as they continuously received vosoritide. We observed a small but significant reduction in achondroplasia score over time in the treated group (Figures 3A-C; c^2^ = 10.1, df = 1, *P* = .0014, b = −0.0011). In Figure 3, we show examples of how individuals’ achondroplasia scores changed over the course of the study. Supplemental Figure 1 shows a variation of Figure 3 with age along the *x*-axis to highlight variation in age at the beginning of treatment. The majority of participants began treatment at 5 years of age (Supplemental Table 1). The left heatmap depicts the first visit achondroplasia score, in which blue denotes regions of the face that are similar to normative, and red regions denote similarity to achondroplasia. The right heatmap shows the observed change in achondroplasia score at the last visit either to a more normative score (blue) or a more achondroplasia-like score (red). We provide paired heatmaps showing the difference in achondroplasia score between first and last visit for each participant in Supplemental Figure 2. The reduction in achondroplasia score effect was not related to differences in age (*P* = .44).

Similarity to the achondroplasia phenotype was not uniform across all facial features; each participant displayed a unique combination of signature achondroplasia facial characteristics before vosoritide treatment. In general, the study cohort did not exhibit prominent frontal bossing. This was evident in the first scan heatmaps (Figure 3, Supplemental Figure 1, leftmost heatmaps) which showed higher normative scores for the forehead and brow ridge across all participants. Almost all participants had the highest achondroplasia scores in the midface, specifically in the orbital and nasal regions, as well as the anteriormost region of the chin. A smaller number of participants also scored high for achondroplasia in the cheeks and lateral temporal and zygomatic regions. The heatmaps of changes in achondroplasia score at the last visit (Figure 3, Supplemental Figure 2, rightmost heatmaps) most often showed responses toward normative facial shape in the orbital region and chin. Some participants also exhibited normative differences in the brow and nasal regions. The last visit heatmaps also showed a shift toward higher achondroplasia scores in certain areas, particularly the cheeks, zygomatic, and orbital regions. In some cases, the nasal region also exhibited an increase in achondroplasia score.

## Discussion

In-depth understanding of the underlying molecular mechanism behind achondroplasia, combined with extensive knowledge of the health implications that extend beyond its physical impact, has paved the way for the development of precision medications that target the root cause of abnormal bone growth. Vosoritide is a synthetic analog CNP that counteracts constitutively activated FGFR3 and the downstream MAPK pathway activity that causes achondroplasia. To investigate the potential benefits of vosoritide, we address complications associated with craniofacial skeletal involvement and use 3D facial imaging to reveal structural changes that may be clinically relevant.

Several facial bones, including the maxilla and zygoma, ossify through the endochondral process. The anatomical consequences of altered ossification of the facial skeleton, which includes the frontal and nasal bones, are respiratory, dental, auditory, and neurological sequelae. As a result, people with achondroplasia are more likely to be affected by sleep-disordered breathing,^6^ recurrent ear infections, dental malocclusion, conductive hearing loss, and craniocervical junction anomalies.^8^^,^^25^^,^^26^

The mandible is composed of 2 hemimandibles, which fuse to form a single bone by the age of 2 years. The maxilla forms the roof of the mouth, the floor of the nasal cavity, and a portion of its lateral wall. It also houses the maxillary sinus and forms part of the inferior rim and floor of the orbits. The 2 maxillary bones join to form the middle third of the face, with the palatine processes of both maxillaries articulating in the midline. The zygoma form the anterior zygomatic arch and the inferior lateral portion of the orbit, whereas the frontal bone forms the anterior part of the cranium, including the frontal sinuses, part of the ethmoid sinuses, and the roof of the nose and orbit. The anterosuperior bony roof of the nasal cavity is formed by the paired nasal bones, which articulate superiorly with the nasal process of the frontal bone and laterally with the frontal process of the maxillary bone.

The involvement of these facial bones in achondroplasia confers anomalies of the airway, causing snoring and obstructive sleep-disordered breathing. Studies have shown that achondroplasia reduces facial depth and shortens the nasal floor and upper pharynx, all of which impact airway morphology through upper airway stenosis. It also causes protrusion of the chin and increases lower facial height. The latter is caused by an increased mandibular plane angle as a result of partial early ossification of cranial bones.^14^, ^15^, ^16^ Sleep disordered breathing is further exacerbated by adenotonsillar hypertrophy and obesity. The impact upon sleep quality can then cause additional adverse effects on growth and development. The disruption to craniofacial growth does not improve with time and may even worsen with age.^16^

Dental anomalies consequent upon a retrognathic maxilla include protrusive maxillary incisors, anterior open bite, and malocclusion, necessitating the need for complex orthodontic management.^4^^,^^12^^,^^13^

Vosoritide, a synthetic CNP analog, has significantly improved long bone growth through its action on the hypertrophic chondrocytes in the cartilaginous growth plate.^11^, ^12^, ^13^ Studies of the use of vosoritide in infants aged 0 to 6 months with achondroplasia also suggests an improvement upon the foramen magnum surface area, as well as facial and sinus volume.^13^

Therefore, we sought to explore the possibility that vosoritide may alter the endochondral ossification process of the facial bones in affected children. We undertook 3D stereophotogrammetry imaging of the participants’ faces recruited at our clinical trial site at baseline and during treatment in the phase 2 and phase 3 studies and compared facial morphology with controls (achondroplasia and normative).

We approached our analysis of the change in shape in 2 ways. First, we tested for total facial shape changes between the first visit and last visit pairs for each of the 14 individuals treated with vosoritide, and we found no significant difference. In our second approach, we used a mixed model to measure the change in achondroplasia score as a response to vosoritide over time, accounting for individual variation. We were able to detect a small negative change in achondroplasia score that was not driven by the effects of age, sex, or treatment protocol (ie, placebo before drug administration vs drug administration throughout). The reduction in achondroplasia score reflects a general change in face shape away from the average features of achondroplasia and toward the average normative face shape after age and sex effects are removed. This approach does not capture the facial shape changes that occurred at the participant level, only those that correspond to the difference between the normative and achondroplasia mean faces. Thus, Supplemental Figure 2 shows a post-hoc visualization of which regions of the face appeared more normative between the first and last visits for all participants.

Although we observed an overall reduction in achondroplasia scores across participants, we also observed significant score variation across sessions. This may be due to differences in participants’ ages when they received vosoritide, resulting in varying treatment responses. Individuals of the same age may have possessed varying receptivity to the drug. Finally, differences in shape caused by imaging and registration variation may have contributed to variability in achondroplasia scores.

When measuring local changes in achondroplasia facial shape scores, we identified several regions that tended to change toward a more normative morphology. The orbital and chin regions showed the most consistent changes toward a more normative score. The nasal region had an inconsistent response, with some participants scoring more normative and others scoring higher for achondroplasia. These changes primarily affected the tip and lateral regions of the nose, rather than the nasal bridge. Although the response to vosoritide resulted in a reduction in full-face achondroplasia score, these results suggest that the most common changes toward a more normative morphology occur around the midface and chin, with less common effects in the cheek and forehead regions.

There are several potential reasons for why were only able to detect significant facial shape changes with the achondroplasia score. First, the inability to detect whole facial shape changes was likely due to our small sample size, which limited our Hotelling’s T2 test to 13 degrees of freedom. As a result, we could only test 13 principal component axes, which explained 85.6% of the total variation in face shape. The pairing of first and last visit for treated individuals also did not account for differing lengths of participation in the study, which included individuals in the study for as long as 6 years or as few as 1.5 years.

Another difference between our hypothesis testing approaches was the specificity of the measurement of facial shape. The achondroplasia score is a single measurement that is defined by the facial shape differences between our normative and achondroplasia control samples, while controlling for the effects of age and sex. It is a more explicit measurement of the achondroplasia phenotype than facial shape change as a whole. Any analysis of full facial shape includes variation that is related to achondroplasia, as well as other significant sources of variation that may obscure small, but clinically meaningful changes in response to treatment. Regression scoring has been demonstrated as an effective way to measure a phenotype of interest for classification between groups, as well as genome wide analysis of facial shape.^27^^,^^28^

Although scoring is a powerful approach to phenotyping, there are differences between our modeling of the achondroplasia facial shape effect and previous efforts by others. Our estimate of the change in achondroplasia score over time includes variation at the level of individuals, which may not align with growth trajectories of achondroplasia shape change with age estimated from cross-sectional data. This could be improved with longitudinal collection of control achondroplasia faces. Morice et al,^16^ using a similar cross-sectional approach, measured 3D cephalometric traits from CT scan data and demonstrated an increase in the magnitude of achondroplasia-related traits in older aged-participants. However, we found no change in achondroplasia score over age. This discrepancy can be caused by differing sources of data: Morice measured 26 craniofacial landmarks on skull and mandible CT scans, whereas we measured soft tissue surfaces with a dense atlas of 5,629 quasilandmarks. We used facial appearance through soft tissue scanning as a proxy for the underlying hard tissues and underdevelopment of endochondral bone.^29^ This is a limitation in that the distribution of fat and muscle also contributes to soft tissue differences, particularly as faces age. We also modeled growth differently, with Maurice regressing log-transformed age onto shape compared with the cubic polynomial approach used in our work here and previously.^20^^,^^23^

Another limitation to consider is the pooling of phase 2 and 3 data. In phase 3, half of the participants received vosoritide from their first visit in the study. In contrast, the other half of the participants received a placebo for 1 year before beginning treatment. In our model, the treatment effect is measured as years from the start of vosoritide treatment; therefore, placebo participants had several shape measurements before they began treatment. We fitted additional fixed effects for treatment protocol and the interaction between protocol and time, and both terms were insignificant (c_protocol_ = .012, *P* = .91; c_protocol:time_ = 2.9, *P* = .087).

### Conclusion

Achondroplasia is a genetic condition caused by constitutively activated FGFR3 signaling, resulting in inhibition of endochondral ossification. This affects not only the long bones, causing disproportionate short stature but also multiple bones in the body conferring many medical complications, some of which are life-threatening. This includes facial bones, such as the maxilla and zygoma, resulting in deformity of the nasopharyngeal airway, respiratory problems, ENT anomalies, a high prevalence of sleep-disordered breathing, and a 50-fold increase in sudden infant death.

Vosoritide is a safe and effective precision treatment for achondroplasia. We sought to explore the potential effects of vosoritide on the facial bones in achondroplasia to determine any potential benefits in the context of these craniofacial anomalies. The results show that facial bone morphology has responded to treatment, further cementing its use in treating complications of achondroplasia other than height. Future longitudinal collection of 3D facial shape for both control and treated individuals would better elucidate the potential for vosoritide to address the craniofacial phenotype seen in achondroplasia, as well as its predictive value for physiological consequences, such as sleep apnea severity and foramen magnum stenosis.

## Data Availability

3D facial surface models for achondroplasia and healthy controls are available through the FaceBase Consortium (http://www.facebase.org/) under accession numbers FB00000491.01 (DOI: https://doi.org/10.25550/VWP) and FB00000861 (DOI: https://doi.org/10.25550/TJ0), respectively.

## Declaration of AI and AI-Assisted Technologies in the Writing Process

No AI tools were used in the preparation of this manuscript at any stage.

## Conflict of Interest

The authors declare no conflicts of interest.

## References

[1] Unger S., Ferreira C.R., Mortier G.R. (2023). Nosology of genetic skeletal disorders: 2023 revision. Am J Med Genet A.

[2] Horton W.A., Hall J.G., Hecht J.T. (2007). Achondroplasia. Lancet.

[3] Shiang R., Thompson L.M., Zhu Y.Z. (1994). Mutations in the transmembrane domain of FGFR3 cause the most common genetic form of dwarfism, achondroplasia. Cell.

[4] Bellus G.A., Hefferon T.W., Ortiz de Luna R.I. (1995). Achondroplasia is defined by recurrent G380R mutations of FGFR3. Am J Hum Genet.

[5] Ornitz D.M., Legeai-Mallet L. (2017). Achondroplasia: development, pathogenesis, and therapy. Dev Dyn.

[6] Stender M., Pimenta J.M., Cheung M., Irving M., Mukherjee S. (2022). Comprehensive literature review on the prevalence of comorbid conditions in patients with achondroplasia. Bone.

[7] Legeai-Mallet L. (2016). C-type natriuretic peptide analog as therapy for achondroplasia. Endocr Dev.

[8] Pauli R.M. (2019). Achondroplasia: a comprehensive clinical review. Orphanet J Rare Dis.

[9] Lorget F., Kaci N., Peng J. (2012). Evaluation of the therapeutic potential of a CNP analog in a FGFR3 mouse model recapitulating achondroplasia. Am J Hum Genet.

[10] Shuhaibar L.C., Kaci N., Egbert J.R. (2021). Phosphatase inhibition by LB-100 enhances BMN-111 stimulation of bone growth. JCI Insight.

[11] Savarirayan R., Irving M., Bacino C.A. (2019). C-type natriuretic peptide analogue therapy in children with achondroplasia. N Engl J Med.

[12] Savarirayan R., Tofts L., Irving M. (2020). Once-daily, subcutaneous vosoritide therapy in children with achondroplasia: a randomised, double-blind, phase 3, placebo-controlled, multicentre trial. Lancet.

[13] Savarirayan R., Wilcox W.R., Harmatz P. (2024). Vosoritide therapy in children with achondroplasia aged 3-59 months: a multinational, randomised, double-blind, placebo-controlled, phase 2 trial. Lancet Child Adolesc Health.

[14] Onodera K., Niikuni N., Chigono T., Nakajima I., Sakata H., Motizuki H. (2006). Sleep disordered breathing in children with achondroplasia. Part 2. Relationship with craniofacial and airway morphology. Int J Pediatr Otorhinolaryngol.

[15] Sato K., Niikuni N., Nakajima I., Shirakawa T., Sakata H. (2007). Surgical treatment for sleep apnea: changes in craniofacial and pharyngeal airway morphology in a child with achondroplasia. J Oral Sci.

[16] Morice A., Taverne M., Eché S. (2023). Craniofacial growth and function in achondroplasia: a multimodal 3D study on 15 patients. Orphanet J Rare Dis.

[17] Pauli R.M., Scott C.I., Wassman E.R. (1984). Apnea and sudden unexpected death in infants with achondroplasia. J Pediatr.

[18] Savarirayan R., Ireland P., Irving M. (2022). International Consensus Statement on the diagnosis, multidisciplinary management and lifelong care of individuals with achondroplasia. Nat Rev Endocrinol.

[19] Duggan S. (2021). Vosoritide: first approval. Drugs.

[20] Hallgrímsson B., Aponte J.D., Katz D.C. (2020). Automated syndrome diagnosis by three-dimensional facial imaging. Genet Med.

[21] Weinberg S.M., Raffensperger Z.D., Kesterke M.J. (2016). The 3D facial norms database: part 1. A web-based craniofacial anthropometric and image repository for the clinical and research community. Cleft Palate Craniofac J.

[22] White J.D., Ortega-Castrillón A., Matthews H. (2019). MeshMonk: open-source large-scale intensive 3D phenotyping. Sci Rep.

[23] Aponte J.D., Bannister J.J., Hoskens H. (2024). An interactive atlas of three-dimensional syndromic facial morphology. Am J Hum Genet.

[24] Matthews H., Vanneste M., Katsura K. (2023). Refining nosology by modelling variation among facial phenotypes: the RASopathies. J Med Genet.

[25] Celenk P., Arici S., Celenk C. (2003). Oral findings in a typical case of achondroplasia. J Int Med Res.

[26] Brewer A.K. (1979). Achondroplasia: general description and dental problems. Quintessence Int Dent Dig.

[27] Vanneste M., Hoskens H., Goovaerts S. (2024). Syndrome-informed phenotyping identifies a polygenic background for achondroplasia-like facial variation in the general population. Nat Commun.

[28] Drake A.G., Klingenberg C.P. (2008). The pace of morphological change: historical transformation of skull shape in St Bernard dogs. Proc Biol Sci.

[29] Young N.M., Sherathiya K., Gutierrez L. (2016). Facial surface morphology predicts variation in internal skeletal shape. Am J Orthod Dentofacial Orthop.

