# Supplementary material for: Three-dimensional craniofacial imaging in children with achondroplasia treated with vosoritide
Source: Genet Med Open. 2025 Oct 13;3:103463. doi: 10.1016/j.gimo.2025.103463 (PMC12670446; doi:10.1016/j.gimo.2025.103463)
Supplement: Supplemental Figure 1 [file mmc1.docx]

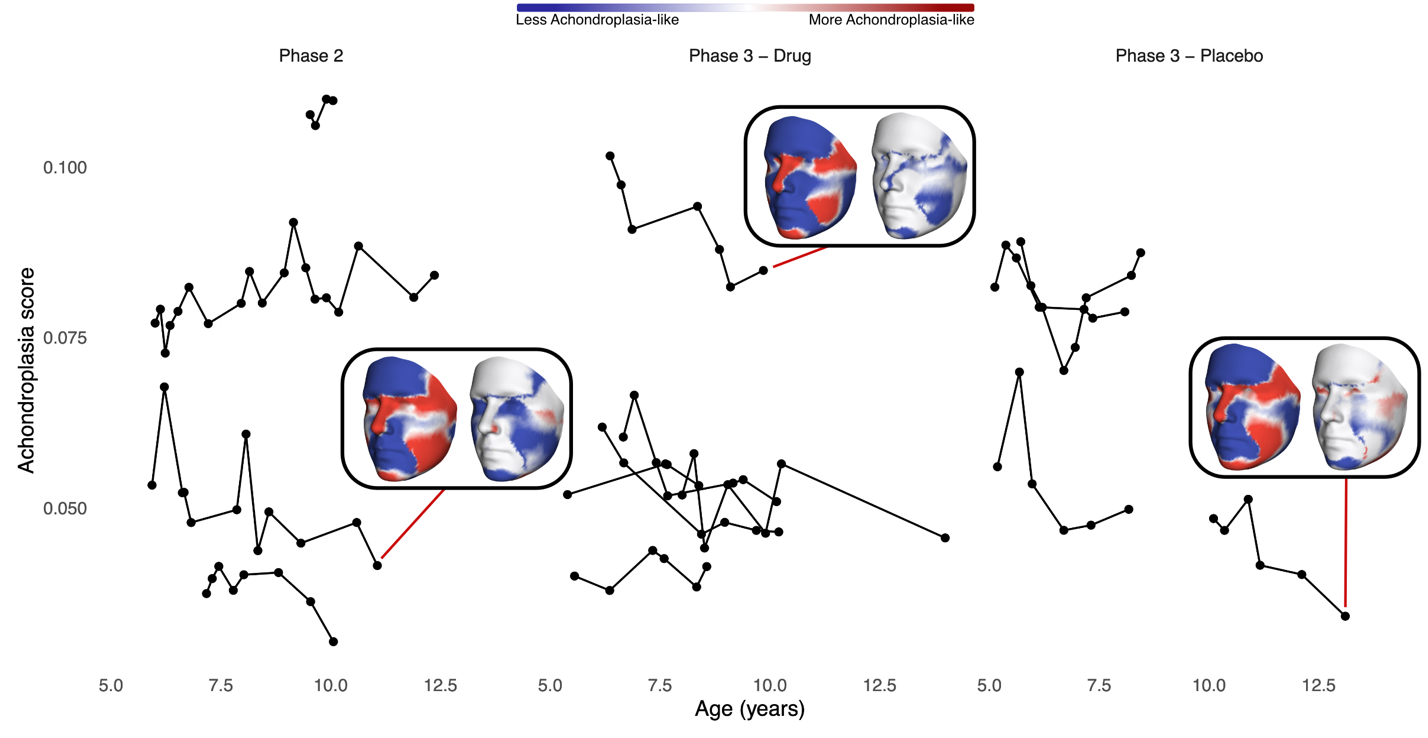


Supplemental Figure 1. Achondroplasia score at each visit for each participant, shown by study phase and by years of age.
