# Supplementary material for: Three-dimensional craniofacial imaging in children with achondroplasia treated with vosoritide
Source: Genet Med Open. 2025 Oct 13;3:103463. doi: 10.1016/j.gimo.2025.103463 (PMC12670446; doi:10.1016/j.gimo.2025.103463)
Supplement: Supplemental Figure 2 [file mmc2.docx]

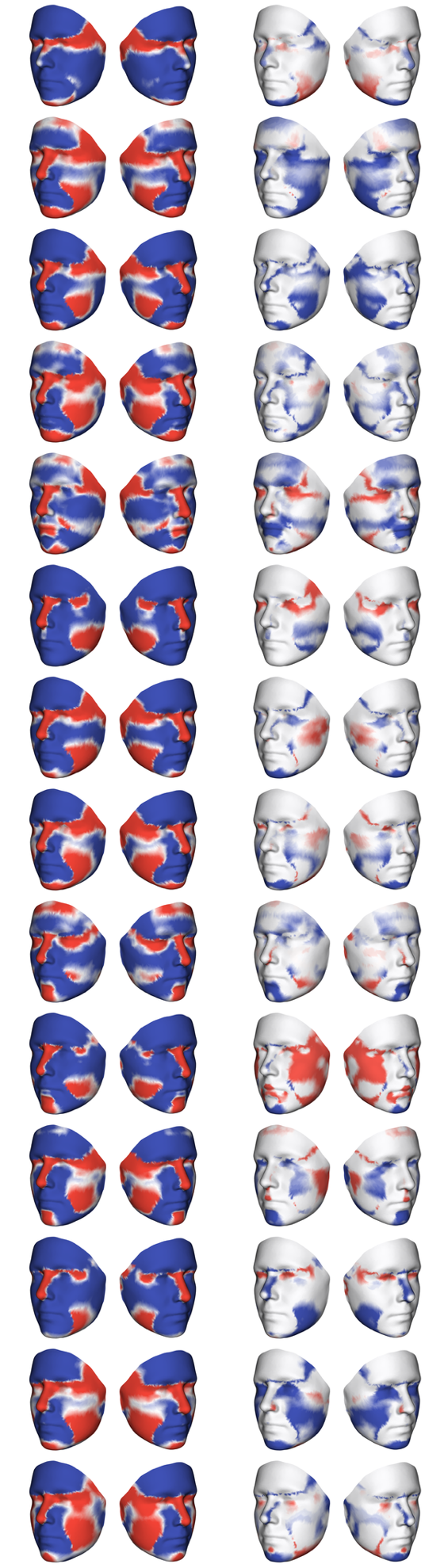


Supplemental Figure 2. Local heatmap achondroplasia scores for all participants. The left two heatmaps show the face at the first visit as a heatmap of achondroplasia scores, with blues signifying regions of the face that are more similar to unaffected controls, and reds signifying similarity to the achondroplasia phenotype. The right two heatmaps show the difference in score at the last visit. Regions in blue score more “unaffected” at the last visit, whereas regions in red score higher for achondroplasia at the last visit.
