# Supplementary material for: Three-dimensional craniofacial imaging in children with achondroplasia treated with vosoritide
Source: Genet Med Open. 2025 Oct 13;3:103463. doi: 10.1016/j.gimo.2025.103463 (PMC12670446; doi:10.1016/j.gimo.2025.103463)
Supplement: Supplemental Table 1 [file mmc3.docx]

| N_ID | Visit | DOV | Phase | Group | Treated | Age | Sex | treatmentLag | Score |
| --- | --- | --- | --- | --- | --- | --- | --- | --- | --- |
| 5 | 1 | 04-07-2018 | 3 | Placebo | 0 | 10.1 | M | -0.7863014 | 0.04842022 |
| 5 | 6 | 02-07-2021 | 3 | Placebo | 1 | 13.1 | M | 2.2109589 | 0.03410591 |
| 5 | 2 | 01-10-2018 | 3 | Placebo | 0 | 10.35 | M | -0.5424658 | 0.04668517 |
| 5 | 3 | 17-04-2019 | 3 | Placebo | 0 | 10.89 | M | 0 | 0.0512518 |
| 5 | 4 | 26-07-2019 | 3 | Placebo | 1 | 11.16 | M | 0.2739726 | 0.04157452 |
| 5 | 5 | 07-07-2020 | 3 | Placebo | 1 | 12.11 | M | 1.22465753 | 0.04021466 |
| 1 | 1 | 28-10-2015 | 2 | Drug | 0 | 7.17 | F | 0 | 0.03742155 |
| 1 | 8 | 20-09-2018 | 2 | Drug | 1 | 10.06 | F | 2.89863014 | 0.03036416 |
| 1 | 2 | 17-12-2015 | 2 | Drug | 1 | 7.3 | F | 0.1369863 | 0.03961871 |
| 1 | 3 | 10-02-2016 | 2 | Drug | 1 | 7.45 | F | 0.28767123 | 0.04141492 |
| 1 | 4 | 08-06-2016 | 2 | Drug | 1 | 7.78 | F | 0.61369863 | 0.03789766 |
| 1 | 5 | 05-09-2016 | 2 | Drug | 1 | 8.02 | F | 0.85753425 | 0.04018384 |
| 1 | 6 | 20-06-2017 | 2 | Drug | 1 | 8.81 | F | 1.64657534 | 0.04050277 |
| 1 | 7 | 14-03-2018 | 2 | Drug | 1 | 9.54 | F | 2.37808219 | 0.03623664 |
| 6 | 1 | 21-02-2018 | 3 | Drug | 0 | 6.36 | M | 0 | 0.10160677 |
| 6 | 7 | 19-08-2021 | 3 | Drug | 1 | 9.85 | M | 3.49315068 | 0.08480664 |
| 6 | 2 | 24-05-2018 | 3 | Drug | 1 | 6.61 | M | 0.25205479 | 0.09735071 |
| 6 | 3 | 22-08-2018 | 3 | Drug | 1 | 6.86 | M | 0.49863014 | 0.09082342 |
| 6 | 4 | 17-02-2020 | 3 | Drug | 1 | 8.35 | M | 1.9890411 | 0.09420747 |
| 6 | 5 | 20-08-2020 | 3 | Drug | 1 | 8.85 | M | 2.49589041 | 0.0878794 |
| 6 | 6 | 19-11-2020 | 3 | Drug | 1 | 9.1 | M | 2.74520548 | 0.08239582 |
| 7 | 1 | 10-09-2018 | 3 | Drug | 0 | 5.39 | M | 0 | 0.05193766 |
| 7 | 4 | 06-09-2021 | 3 | Drug | 1 | 8.38 | M | 2.99178082 | 0.05326428 |
| 7 | 2 | 02-12-2020 | 3 | Drug | 1 | 7.62 | M | 2.23013699 | 0.05643715 |
| 7 | 3 | 16-12-2020 | 3 | Drug | 1 | 7.66 | M | 2.26849315 | 0.05633138 |
| 8 | 1 | 10-09-2018 | 3 | Drug | 0 | 5.55 | F | 0 | 0.03999456 |
| 8 | 6 | 15-09-2021 | 3 | Drug | 1 | 8.56 | F | 3.01643836 | 0.04138623 |
| 8 | 2 | 01-07-2019 | 3 | Drug | 1 | 6.35 | F | 0.80547945 | 0.03787457 |
| 8 | 3 | 22-06-2020 | 3 | Drug | 1 | 7.33 | F | 1.78356164 | 0.04374718 |
| 8 | 4 | 28-09-2020 | 3 | Drug | 1 | 7.59 | F | 2.05205479 | 0.04255495 |
| 8 | 5 | 23-06-2021 | 3 | Drug | 1 | 8.33 | F | 2.78630137 | 0.03838596 |
| 2 | 1 | 21-07-2014 | 2 | Drug | 0 | 9.53 | M | 0 | 0.10765917 |
| 2 | 4 | 28-01-2015 | 2 | Drug | 1 | 10.05 | M | 0.52328767 | 0.10971309 |
| 2 | 2 | 03-09-2014 | 2 | Drug | 1 | 9.65 | M | 0.12054795 | 0.10605528 |
| 2 | 3 | 03-12-2014 | 2 | Drug | 1 | 9.9 | M | 0.36986301 | 0.10993756 |
| 3 | 1 | 05-08-2014 | 2 | Drug | 0 | 6 | M | 0 | 0.07708525 |
| 3 | 19 | 15-12-2020 | 2 | Drug | 1 | 12.36 | M | 6.36712329 | 0.08410521 |
| 3 | 9 | 29-09-2016 | 2 | Drug | 1 | 8.15 | M | 2.15342466 | 0.08465883 |
| 3 | 10 | 12-01-2017 | 2 | Drug | 1 | 8.44 | M | 2.44109589 | 0.08003282 |
| 3 | 11 | 13-07-2017 | 2 | Drug | 1 | 8.94 | M | 2.93972603 | 0.08445949 |
| 3 | 12 | 28-09-2017 | 2 | Drug | 1 | 9.15 | M | 3.15068493 | 0.09185734 |
| 3 | 13 | 08-01-2018 | 2 | Drug | 1 | 9.43 | M | 3.43013699 | 0.08518962 |
| 3 | 14 | 28-03-2018 | 2 | Drug | 1 | 9.64 | M | 3.64657534 | 0.08059431 |
| 3 | 15 | 28-06-2018 | 2 | Drug | 1 | 9.9 | M | 3.89863014 | 0.08079902 |
| 3 | 16 | 08-10-2018 | 2 | Drug | 1 | 10.18 | M | 4.17808219 | 0.07868277 |
| 3 | 17 | 21-03-2019 | 2 | Drug | 1 | 10.63 | M | 4.62739726 | 0.08838937 |
| 3 | 18 | 26-06-2020 | 2 | Drug | 1 | 11.89 | M | 5.89589041 | 0.08085573 |
| 3 | 2 | 17-09-2014 | 2 | Drug | 1 | 6.12 | M | 0.11780822 | 0.07912949 |
| 3 | 3 | 27-10-2014 | 2 | Drug | 1 | 6.23 | M | 0.22739726 | 0.07268603 |
| 3 | 4 | 08-12-2014 | 2 | Drug | 1 | 6.34 | M | 0.34246575 | 0.07674328 |
| 3 | 5 | 09-02-2015 | 2 | Drug | 1 | 6.52 | M | 0.51506849 | 0.07881102 |
| 3 | 6 | 12-05-2015 | 2 | Drug | 1 | 6.77 | M | 0.76712329 | 0.08233502 |
| 3 | 7 | 21-10-2015 | 2 | Drug | 1 | 7.21 | M | 1.2109589 | 0.07700534 |
| 3 | 8 | 21-07-2016 | 2 | Drug | 1 | 7.96 | M | 1.96164384 | 0.07998348 |
| 9 | 1 | 15-08-2018 | 3 | Placebo | 0 | 5.18 | F | -0.7917808 | 0.05602064 |
| 9 | 6 | 11-08-2021 | 3 | Placebo | 1 | 8.17 | F | 2.2 | 0.04977911 |
| 9 | 2 | 12-02-2019 | 3 | Placebo | 0 | 5.68 | F | -0.2958904 | 0.06991259 |
| 9 | 3 | 31-05-2019 | 3 | Placebo | 0 | 5.97 | F | 0 | 0.05350899 |
| 9 | 4 | 17-02-2020 | 3 | Placebo | 1 | 6.69 | F | 0.71780822 | 0.04670068 |
| 9 | 5 | 30-09-2020 | 3 | Placebo | 1 | 7.31 | F | 1.3369863 | 0.04745101 |
| 10 | 1 | 04-04-2018 | 3 | Drug | 0 | 8 | F | 0 | 0.05188019 |
| 10 | 7 | 01-04-2024 | 3 | Drug | 1 | 13.99 | F | 5.99726027 | 0.04558251 |
| 10 | 2 | 11-07-2018 | 3 | Drug | 1 | 8.27 | F | 0.26849315 | 0.0579428 |
| 10 | 3 | 08-10-2018 | 3 | Drug | 1 | 8.51 | F | 0.51232877 | 0.04410308 |
| 10 | 4 | 17-04-2019 | 3 | Drug | 1 | 9.04 | F | 1.03561644 | 0.05338983 |
| 10 | 5 | 29-02-2020 | 3 | Drug | 1 | 9.9 | F | 1.90684932 | 0.04626711 |
| 10 | 6 | 08-07-2020 | 3 | Drug | 1 | 10.26 | F | 2.2630137 | 0.05644492 |
| 11 | 1 | 20-07-2018 | 3 | Placebo | 0 | 5.12 | M | -0.4931507 | 0.0823619 |
| 11 | 7 | 07-07-2021 | 3 | Placebo | 1 | 8.08 | M | 2.4739726 | 0.07874445 |
| 11 | 2 | 19-10-2018 | 3 | Placebo | 0 | 5.37 | M | -0.2438356 | 0.08852549 |
| 11 | 3 | 16-01-2019 | 3 | Placebo | 0 | 5.61 | M | 0 | 0.08664278 |
| 11 | 4 | 26-07-2019 | 3 | Placebo | 1 | 6.13 | M | 0.52328767 | 0.07941541 |
| 11 | 5 | 02-08-2020 | 3 | Placebo | 1 | 7.15 | M | 1.54520548 | 0.07910449 |
| 11 | 6 | 14-10-2020 | 3 | Placebo | 1 | 7.35 | M | 1.74520548 | 0.07780844 |
| 12 | 1 | 14-02-2018 | 3 | Placebo | 0 | 5.71 | M | -0.9780822 | 0.08902002 |
| 12 | 8 | 05-11-2020 | 3 | Placebo | 1 | 8.44 | M | 1.74794521 | 0.08741077 |
| 12 | 2 | 09-05-2018 | 3 | Placebo | 0 | 5.94 | M | -0.7479452 | 0.08257945 |
| 12 | 3 | 10-08-2018 | 3 | Placebo | 0 | 6.2 | M | -0.4931507 | 0.07940269 |
| 12 | 4 | 06-02-2019 | 3 | Placebo | 0 | 6.69 | M | 0 | 0.07014008 |
| 12 | 5 | 10-05-2019 | 3 | Placebo | 1 | 6.95 | M | 0.25479452 | 0.07352539 |
| 12 | 6 | 09-08-2019 | 3 | Placebo | 1 | 7.2 | M | 0.50410959 | 0.08079985 |
| 12 | 7 | 20-08-2020 | 3 | Placebo | 1 | 8.23 | M | 1.5369863 | 0.08408058 |
| 13 | 1 | 10-01-2018 | 3 | Drug | 0 | 6.66 | M | 0 | 0.06037568 |
| 13 | 7 | 08-07-2021 | 3 | Drug | 1 | 10.15 | M | 3.49315068 | 0.05090948 |
| 13 | 2 | 11-04-2018 | 3 | Drug | 1 | 6.91 | M | 0.24931507 | 0.06651583 |
| 13 | 3 | 10-10-2018 | 3 | Drug | 1 | 7.41 | M | 0.74794521 | 0.05662942 |
| 13 | 4 | 14-01-2019 | 3 | Drug | 1 | 7.67 | M | 1.0109589 | 0.05176098 |
| 13 | 5 | 10-07-2020 | 3 | Drug | 1 | 9.16 | M | 2.49863014 | 0.05363004 |
| 13 | 6 | 01-10-2020 | 3 | Drug | 1 | 9.39 | M | 2.7260274 | 0.05412801 |
| 4 | 1 | 28-10-2015 | 2 | Drug | 0 | 5.93 | M | 0 | 0.05332604 |
| 4 | 12 | 15-12-2020 | 2 | Drug | 1 | 11.06 | M | 5.1369863 | 0.04152962 |
| 4 | 9 | 28-06-2018 | 2 | Drug | 1 | 8.59 | M | 2.66849315 | 0.04940363 |
| 4 | 10 | 21-03-2019 | 2 | Drug | 1 | 9.32 | M | 3.39726027 | 0.04481427 |
| 4 | 11 | 26-06-2020 | 2 | Drug | 1 | 10.59 | M | 4.66575342 | 0.04784299 |
| 4 | 2 | 10-02-2016 | 2 | Drug | 1 | 6.21 | M | 0.28767123 | 0.06772932 |
| 4 | 3 | 08-07-2016 | 2 | Drug | 1 | 6.62 | M | 0.69589041 | 0.05220609 |
| 4 | 4 | 21-07-2016 | 2 | Drug | 1 | 6.66 | M | 0.73150685 | 0.05226717 |
| 4 | 5 | 20-09-2016 | 2 | Drug | 1 | 6.82 | M | 0.89863014 | 0.04783716 |
| 4 | 6 | 02-10-2017 | 2 | Drug | 1 | 7.86 | M | 1.93150685 | 0.0497143 |
| 4 | 7 | 19-12-2017 | 2 | Drug | 1 | 8.07 | M | 2.14520548 | 0.06080473 |
| 4 | 8 | 29-03-2018 | 2 | Drug | 1 | 8.34 | M | 2.41917808 | 0.04371882 |
| 14 | 1 | 13-12-2017 | 3 | Drug | 0 | 6.18 | F | 0 | 0.0618393 |
| 14 | 6 | 20-12-2021 | 3 | Drug | 1 | 10.2 | F | 4.02191781 | 0.04646035 |
| 14 | 2 | 12-06-2018 | 3 | Drug | 1 | 6.67 | F | 0.49589041 | 0.05658817 |
| 14 | 3 | 18-03-2020 | 3 | Drug | 1 | 8.44 | F | 2.2630137 | 0.04613218 |
| 14 | 4 | 30-09-2020 | 3 | Drug | 1 | 8.97 | F | 2.8 | 0.04786605 |
| 14 | 5 | 17-06-2021 | 3 | Drug | 1 | 9.69 | F | 3.51232877 | 0.04668234 |

Supplemental table 1. Demographics of all participants for all visits included in the study.
